# Prevalence of musculoskeletal injuries and associated risk factors in Brazilian esports players: a cross-sectional study

**DOI:** 10.1038/s41598-026-41487-2

**Published:** 2026-03-07

**Authors:** Cassius Iury Anselmo-e-Silva, Aldair Darlan Santos-de-Araújo, Maria Julia Checo Melger, Hygor Ferreira-Silva, Décio Bueno Neto, Vitor Kenji Issi, Wilson Tenório de Almeida Filho, Wilton Martins dos Santos Junior, Natanael Teixeira Alves de Sousa

**Affiliations:** 1 Physical Therapy Department, Federal University of São Carlos, São Carlos, SP Brazil; 2 Department of Physical Therapy, Central University of Tiradentes, Maceió, AL Brazil; 3 Cinemática Sport and Health, R. OlindinaCampos Teixeira, Maceió, Al 57036-690 Brazil; 4 Kenji E-Sports, São Paulo, SP Brazil

**Keywords:** Musculoskeletal injury, Video gaming, Epidemiology, E-Sports, Diseases, Health care, Health occupations, Medical research, Risk factors

## Abstract

Electronic sports (esports) are competitive video game activities with varying physical demands depending on the game modality. Despite their growing popularity, limited research has addressed the prevalence and risk factors of musculoskeletal injuries in this population. This cross-sectional observational study aimed to determine the prevalence and distribution of injuries by body region in Brazilian esports players and to identify associations with injury occurrence. A total of 365 players completed an online form covering sociodemographic information, duration of esports practice, daily hours played, and injury history over the previous 12 months. Statistical analyses included descriptive measures, group comparisons (t-test, Mann-Whitney, Chi-square), binary and multiple logistic regression, and Receiver Operating Characteristic (ROC) curve analysis. Among participants, 318 (87.12%) were male, and 113 (30.96%) reported injuries within the past year. The upper limb was the most commonly affected region, with the wrist accounting for 28.49% of reported injuries. Multiple logistic regression revealed significant associations between injury occurrence and both years of practice (OR = 1.110; *p* = 0.005) and weekly practice frequency (OR = 1.183; *p* = 0.032). Although the ROC curve showed limited discriminative ability, these factors were linked to higher wrist injury risk. Preventive strategies should consider training volume and practice frequency.

## Introduction

The term electronic sports (esports) refers to competitive video games, whether professional or amateur, in which individual players or teams engage in activities mediated by electronic systems^1^. Esports are understood as organized matches or competitions involving video games, as they share structural elements similar to those of traditional sports, including the presence of athletes, spectators, championships, and professional leagues^2,3^. However, the National Collegiate Athletic Association (NCAA) has frequently debated the possibility of recognizing esports as a sport^4^.

The rapid expansion of the esports industry, along with the continuous growth of its player and viewer base, has been accompanied by a notable increase in health-related issues reported by esports athletes^5,6^. It is well established that sedentary behavior, repetitive postural movements, and frequent use of hand muscles are inherent to the lifestyle of this population^7,8^. Consequently, physical inactivity may contribute to the development of musculoskeletal pain among esports players^9^, while prolonged video game use may also increase the risk of repetitive strain injuries^10^. However, there remains a scarcity of studies that thoroughly examine the long-term health implications of these risk factors in this population^11^.

In addition to the potential risk factors associated with the development of musculoskeletal injuries in esports players, identifying the specific game genre practiced is essential, as different types of games impose distinct physical demands^12^. Esports encompass a wide range of modalities organized into distinct categories, such as sports simulation games (e.g., FIFA EA Sports^®^), First-Person Shooters (e.g., Call of Duty, Counter-Strike), Battle Royale games (e.g., Fortnite), Strategy games (e.g., StarCraft), and Multiplayer Online Battle Arena (MOBA) titles (e.g., League of Legends)^13^. Thus, the distinct physical demands associated with each game category may directly influence players’ kinematic patterns^12^, potentially serving as a risk factor for musculoskeletal injuries. In this context, ergonomic, biological, and psychosocial risk factors are also considered, as their combined effects may significantly impact the daily lives of esports practitioners, similarly to the risks observed in other sports disciplines, recreational activities, and occupational environments^14^.

Given this scenario and the continued growth of esports participation, identifying the prevalence and potential risk factors for musculoskeletal injuries is crucial for guiding health promotion and injury prevention strategies in esports practitioners. Although injuries related to esports participation have already been acknowledged in the literature, there is still a lack of comprehensive studies investigating the underlying risk factors for musculoskeletal disorders, as well as the most commonly affected body regions. In this context, the primary aim is to analyze the prevalence of injuries, considering their distribution across body regions, in esports players in Brazil. The secondary objective is to explore and identify factors associated with injury development and discriminate the occurrence of injuries by body region. Our hypothesis is that the prevalence of musculoskeletal injuries is higher in body regions that are more intensively involved in the specific demands of esports practice. Additionally, we hypothesize that variables such as age, practice time, weekly frequency, and daily duration of esports gameplay may have discriminative power in identifying musculoskeletal injuries.

## Methods

### Study design and ethical procedures

This is a cross-sectional study conducted following the recommendations of Checklist for Reporting Results of Internet E-Surveys (CHERRIES)^15^ e Strengthening the Reporting of Observational Studies in Epidemiology (STROBE statement)^16^. This study was approved by Tiradentes University Center Human Ethics Committee (protocol number: 02704218.6.0000.5641) and complied with the Declaration of Helsinki. All individuals who voluntarily agreed to participate were thoroughly informed about the purpose of the study and provided written informed consent prior to their inclusion in the study. Data collection took place between November 2019 to November 2020.

### Participants

We conducted an open survey with data collected through an online form. For this study, Brazilian esports players were included with at least ≥ 12 months of experience (practice time) in the electronic game modalities.

Participants were recruited through social media advertisements and during esports competitions. The sample consisted of volunteers selected by convenience^17,18^.

Brazilian esports players of both sexes, aged 18 years or older, residing in Brazil, regardless of the game modality practiced and with at least ≥ 12 months of experience, were included in the study. Participation required full completion of an online form, which could be self-administered remotely or applied in person by a researcher during events and competitions. All participants were required to provide informed consent prior to participation. Participants who did not complete the form, provided inconsistent or duplicate responses, or did not give formal consent were excluded. Responses from individuals who did not identify as esports players at the time of data collection were also disregarded.

### Procedures

Data was collected using an online survey registered on Google Forms. The form items were drafted by a physiotherapist with experience in the field (NTAS) and subsequently reviewed and approved by the research team. To check the clarity and comprehension of the questions, which were presented in Brazilian Portuguese, a pilot test was carried out before the main data collection process began^19^.

Upon accessing the online questionnaire link, participants were first directed to a page containing the Informed Consent Form, which outlined the study’s objectives and procedures. Only after agreeing to the terms were they allowed to proceed to the questionnaire itself. Once the questionnaire was fully completed, the responses were automatically send by e-mail to the principal investigator for registration and analysis.

The questionnaire was divided into three main sections. The first section addressed participants’ sociodemographic characteristics, including questions about age (in years) and sex. The second section collected information related to esports participation, including the following questions: “How long have you been playing esports?”; “On average, how many hours per day do you play?”; and “How many days per week do you usually play esports?”. The third section focused on the occurrence of musculoskeletal injuries associated with esports participation, through the following questions: “Have you experienced any musculoskeletal injury related to esports practice in the past 12 months?” (Responses: yes or no); “How many injuries have you had during your time practicing esports?”; “If injured, which part(s) of the body were affected?”; and “Do you receive regular guidance on injury prevention? If yes, from which professional(s)?”. For the questions related to musculoskeletal injuries, participants were instructed to report any musculoskeletal complaint or injury that resulted in absence from esports practice or competition (time-loss)^20,21^. For data analysis, only time-loss injuries were considered.In total, the questionnaire consisted of nine questions.

### Statistical analysis

Data were presented as mean ± standard deviation (SD), median and interquartile range (IQR25-75), or as absolute values and percentages (%), depending on the data distribution. The Shapiro–Wilk test was used to assess the normality of continuous variables. For comparison between groups (men vs. female), the independent *t*-test was applied for normally distributed variables, while the Mann-Whitney *U* test was used when the assumption of normality was violated. Categorical variables were compared using the Chi-square test.

To identify predictors of injury occurrence among esports players (yes or no), a simple binary logistic regression analysis was performed. Variables with a p-value < 0.20 in the univariate analysis were entered into a multiple logistic regression model using the forward selection method^22^. The final model was refined using the enter method to improve the explanation of variance (R^2^ Cox & Snell and R^2^ Nagelkerke). Results were reported as odds ratios (OR) with 95% confidence intervals (95% CI). Multicollinearity was assessed using Variance Inflation Factor (VIF) and Tolerance statistics. All VIF values were below 5 and all Tolerance values were above 0.2, indicating the absence of multicollinearity among the independent variables^23^. The goodness-of-fit of the logistic regression model was assessed using the Hosmer–Lemeshow test, a p-value greater than 0.05 indicates no evidence of poor fit, suggesting that the model adequately fits the data^24^.

Receiver Operating Characteristic (ROC) curve analysis was used to evaluate the discriminative power of age, practice time, weekly frequency, and daily hours in identifying musculoskeletal injuries^25^. Area under the curve (AUC) values were classified as follows: AUC < 0.5, low predictive capacity; 0.7 ≤ AUC < 0.8, good predictive capacity; and 0.8 ≤ AUC < 0.9, excellent predictive capacity. Cutoff points were established only when a good or excellent predictive capacity was observed (AUC ≥ 0.70). When the AUC value did not reach this threshold, cutoff points were not defined^26^.

The AUC was analyzed using the GraphPad Prism version 8.0.1 for Windows (GraphPad Software, Boston, Massachusetts USA), < www.graphpad.com >. For other analyses were conducted using the Statistical Package for the Social Sciences (SPSS) [IBM—SPSS, version 20.0 for Windows, Armonk, NY, <https://www.ibm.com/br-pt%3E/]. The probability of type 1 error occurrence was established at 5% for all tests (*p* < 0.05).

## Results

A total of 365 participants comprised the final sample, the majority of whom were male (87.12%), with a mean age of 20.48 ± 3.37 years. Regarding the variable “hours played per day”, males reported a significantly higher number of hours played compared to females (*p* = 0.009). Additionally, no statistically significant difference was observed between sexes in relation to the number of self-reported injuries (29.87% in males and 38.29% in females; *p* = 0.224), as well as for the other analyzed variables (*p* > 0.05) (Table 1).

**Table 1:** Characteristics of participants (*N* = 365).

Variables	All volunteers (*n* = 365)	Men (*n* = 318)	Female (*n* = 47)	*P* value
Age (years)	20 (18–22)	20.48 ± 3.37	22.72 ± 4.49	< 0.001*
18–24	325 (89.04)	290 (91.19)	35 (74.46)	0.002*
25–29	28 (7.67)	20 (6.28)	8 (17.02)	
30–34	10 (2.74)	7 (2.20)	3 (6.38)	
40–44	1 (0.27)	0 (0.00)	1 (2.12)	
45–49	1 (0.27)	1 (0.31)	0 (0.00)	
Practice time (years)	6 (4–8)	6 (4–8)	5 (2–7)	0.624
Hours played (per day)	5 (3–7)	5.83 ± 3.38	4.63 ± 3.03	0.009*
Weekly practice frequency (times per week)	6 (4–8)	7 (5–7)	6 (4–7)	0.569
Injury (12 months)				
Yes	113 (30.95)	95 (29.87)	18 (38.29)	0.224
No	252 (69.04)	223 (70.12)	29 (61.70)	
Number of E-sports-related injuries	1 (0–2)	1 (0–2)	1 (0–2)	0.148
Professional guidance on injury prevention				
Physician	137 (37.53)	121 (38.05)	16 (34.04)	0.596
Physiotherapist	137 (37.53)	123 (38.67)	14 (29.78)	0.240
Physical Education Professional	149 (40.82)	135 (42.45)	14 (29.78)	0.099
No guidance	150 (41.09)	126 (39.62)	24 (51.06)	0.137

Data in mean ± standard deviation, median and interquartile range (IQR25-75) or absolute value and percentage (%). *Statistical significance for comparison between groups (*p* < 0.05).

### Distribution of injuries by body region

In the overall sample, the most frequently self-reported injury sites were the wrist (28.49%), low back (20.00%), hands (18.35%), and fingers (15.89%). When comparing males and females, no statistically significant differences were observed for any body region (*p* > 0.05). Among the most prevalent injury sites, similar odds were found between sexes for wrist injuries (OR = 1.505; 95% CI: 0.790–2.865), low back injuries (OR = 1.444; 95% CI: 0.708–2.946), and finger injuries (OR = 0.916; 95% CI: 0.389–2.159) (Table 2).

**Table 2:** Distribution of injuries by body region in Esports.

Body region	All volunteers (*n* = 365)	Men (*n* = 318)	Female (*n* = 47)	Odds ratio (CI95%)	*P* value
Shoulder	35 (9.58)	30 (9.43)	5 (10.63)	1.143 (0.420–3.108)	0.794
Arm	13 (3.56)	12 (3.77)	1 (2.12)	0.554 (0.070–4.364)	0.570
Elbow	14 (3.83)	12 (3.77)	2 (4.25)	1.133 (0.246–5.230)	0.872
Forearm	15 (4.11)	14 (4.40)	1 (2.12)	0.472 (0.061–3.675)	0.463
Wrist	104 (28.49)	87 (27.35)	17 (36.17)	1.505 (0.790–2.865)	0.212
Hand	67 (18.35)	57 (17.920	10 (21.27)	1.238 (0.582–2.633)	0.580
Fingers	58 (15.89)	51 (16.03)	7 (14.89)	0.916 (0.389–2.159)	0.841
Neck	54 (14.79)	49 (15.40)	5 (10.63)	0.654 (0.246–1.734)	0.390
Low back	73 (20.00)	61 (19.18)	12 (25.53)	1.444 (0.708–2.946)	0.310

Data in absolute value and percentage (%): CI.: confidence interval. No statistically significant differences for comparisons between groups.

### Risk factors associated with injury occurrence

Table 3 presents the different simple binary logistic regression models potentially associated with injury risk by body region among esports players. A statistically significant association was observed between practice time (in years) (β = 0.114; OR = 1.120; *p* = 0.002) and weekly frequency of practice (β = 0.161; OR: 1.175; *p* = 0.036) with shoulder injury. Hours played (per day) (β = 0.078; OR: 1.081; *p* = 0.049) were significantly associated with neck injury. Additionally, practice time (in years) (β = 0.100; OR: 1.105; *p* < 0.011) were significantly associated with low back injury.

**Table 3:** Binary logistic regression analysis of factors potentially associated with injuries in Esports.

Variables	β	S.E.	Wald	*P* value	Odds ratio (CI95%)	*R*^2^ Cox & Snell	*R*^2^ Nagelkerke
Shoulder							
Age (years)	– 0.024	0.054	0.195	0.659	0.976 (0.878, 1.086)	0.001	0.001
Sex (0. female; 1. male)	– 0.134	0.511	0.068	0.794	0.875 (0.322, 2.380)	0.000	0.000
Practice time (years)	0.072	0.052	1.897	0.168^†^	1.074 (0.970, 1.190)	0.005	0.011
Hours played (per day)	0.080	0.046	3.032	0.082^†^	1.084 (0.990, 1.186)	0.008	0.016
Weekly practice frequency	0.093	0.116	0.638	0.424	1.097 (0.874, 1.378)	0.002	0.004
Arm							
Age (years)	– 0.036	0.091	0.154	0.694	0.965 (0.806, 1.154)	0.000	0.002
Sex (0. female; 1. male)	0.590	1.053	0.314	0.575	1.804 (0.229, 14.202)	0.001	0.004
Practice time (years)	0.057	0.083	0.468	0.494	1.058 (0.900, 1.244)	0.001	0.005
Hours played (per day)	0.062	0.073	0.728	0.394	1.064 (0.922, 1.229)	0.002	0.007
Weekly practice frequency	0.006	0.174	0.001	0.970	1.006 (0.716, 1.416)	0.000	0.000
Elbow							
Age (years)	– 0.184	0.134	1.885	0.170^†^	0.832 (0.640, 1.082)	0.007	0.026
Sex (0. female; 1. male)	– 0.125	0.780	0.026	0.873	0.882 (0.191, 4.072)	0.000	0.000
Practice time (years)	– 0.100	0.091	1.210	0.271	0.905 (0.757, 1.081)	0.004	0.013
Hours played (per day)	0.089	0.067	1.754	0.185^†^	1.093 (0.958, 1.247)	0.004	0.015
Weekly practice frequency	0.075	0.177	0.179	0.672	1.078 (0.762, 1.525)	0.001	0.002
Forearm							
Age (years)	– 0.076	0.098	0.602	0.438	0.927 (0.765, 1.123)	0.002	0.007
Sex (0. female; 1. male)	0.751	1.047	0.514	0.473	2.118 (0.272, 16.494)	0.002	0.006
Practice time (years)	0.077	0.076	1.030	0.310	1.080 (0.931, 1.254)	0.003	0.009
Hours played (per day)	0.054	0.070	0.593	0.441	1.055 (0.920, 1.210)	0.002	0.005
Weekly practice frequency	0.110	0.176	0.387	0.534	1.116 (0.790, 1.577)	0.001	0.004
Wrist							
Age (years)	0.060	0.031	3.737	0.053^†^	1.061 (0.999, 1.127)	0.010	0.015
Sex (0. female; 1. male)	– 0.409	0.329	1.546	0.214	0.665 (0.349, 1.266)	0.004	0.006
Practice time (years)	0.114	0.036	10.011	0.002*^†^	1.120 (1.044, 1.202)	0.028	0.040
Hours played (per day)	0.031	0.034	0.840	0.359	1.031 (0.966, 1.102)	0.002	0.003
Weekly practice frequency	0.161	0.077	4.402	0.036*^†^	1.175 (1.011, 1.365)	0.013	0.018
Hand							
Age (years)	– 0.043	0.043	0.988	0.320	0.958 (0.880, 1.043)	0.003	0.005
Sex (0. female; 1. male)	– 0.213	0.385	0.306	0.580	0.808 (0.380, 1.719)	0.001	0.001
Practice time (years)	0.024	0.041	0.351	0.554	1.025 (0.946, 1.110)	0.001	0.002
Hours played (per day)	0.061	0.037	2.632	0.105^†^	1.062 (0.987, 1.143)	0.007	0.011
Weekly practice frequency	0.040	0.085	0.225	0.635	1.041 (0.882, 1.229)	0.001	0.001
Fingers							
Age (years)	– 0.052	0.048	1.200	0.273	0.949 (0.865, 1.042)	0.004	0.006
Sex (0. female; 1. male)	0.088	0.437	0.040	0.841	1.091 (0.463, 2.572)	0.000	0.000
Practice time (years)	0.026	0.043	0.363	0.547	1.026 (0.943, 1.117)	0.001	0.002
Hours played (per day)	0.059	0.039	2.285	0.131^†^	1.061 (0.983, 1.146)	0.006	0.010
Weekly practice frequency	0.014	0.088	0.025	0.874	1.014 (0.853, 1.206)	0.000	0.000
Neck							
Age (years)	– 0.025	0.045	0.308	0.579	0.975 (0.894, 1.065)	0.001	0.002
Sex (0. female; 1. male)	0.425	0.498	0.730	0.393	1.530 (0.577, 4.060)	0.002	0.004
Practice time (years)	0.021	0.045	0.220	0.639	1.021 (0.936, 1.114)	0.001	0.001
Hours played (per day)	0.078	0.039	3.874	0.049*^†^	1.081 (1.000, 1.168)	0.010	0.018
Weekly practice frequency	0.091	0.096	0.898	0.343	1.095 (0.908, 1.321)	0.003	0.005
Low back							
Age (years)	– 0.032	0.040	0.646	0.421	0.968 (0.895, 1.048)	0.002	0.003
Sex (0. female; 1. male)	– 0.368	0.364	1.023	0.312	0.692 (0.339, 1.412)	0.003	0.004
Practice time (years)	0.100	0.039	6.471	0.011*^†^	1.105 (1.023, 1.194)	0.018	0.028
Hours played (per day)	0.045	0.037	1.500	0.221	1.046 (0.973, 1.125)	0.004	0.006
Weekly practice frequency	0.107	0.085	1.570	0.210	1.113 (0.942, 1.315)	0.004	0.007

β: beta; kg: kilos; m: meter; BMI: body mass index; S.E.: standard error; %: percentage; C.I.: confidence interval. *Statistical significance for binary logistic regression analysis (*p* < 0.05); ^†^Statistical significance for multiple regression analysis (*p* < 0.020).

### Multiple logistic regression

A multiple logistic regression analysis was conducted to identify factors associated with injury occurrence by body region (Table 4). Practice time (in years) and weekly practice frequency were significantly associated with wrist injury. Practice time had a regression coefficient of β = 0.104 (*p* = 0.005), indicating that each additional year of esports experience was associated with an approximately 11% increase in the odds of injury (OR = 1.110; IC95%: 1.033–1.192). Weekly practice frequency showed a coefficient of β = 0.168 (*p* = 0.032), suggesting that each additional day of practice per week was associated with an approximately 18.3% increase in the odds of injury (OR = 1.183; IC95%: 1.015–1.379). However, the model explained only 4.7% to 6.7% of the variance in wrist injury over the past 12 months (R² Cox & Snell’s = 0.047; R² Nagelkerke = 0.067). Although both practice time and weekly frequency were statistically significantly associated with the outcome, the model accounted for only a small portion of the variance in the dependent variable.

**Table 4:** Binary logistic regression analysis of factors potentially associated with injuries in Esports.

Model	Variables	β	S.E.	Wald	*P* value	Odds ratio (CI95%)	*R*^2^ Cox & Snell	*R*^2^ Nagelkerke	Hosmer and Lemeshow
Shoulder									
1	Practice time (years)	0.058	0.053	1.199	0.274	1.060 (0.955, 1.176)	0.011	0.023	0.638
	Hours played (per day)	0.072	0.047	2.311	0.128	1.074 (0.979, 1.178)			
	Intercept	– 3.050	0.464	43.287	< 0.001*	0.047			
Elbow									
1	Age (years)	– 0.161	0.133	1.475	– 0.161	0.133 (0.657, 1.104)	0.010	0.035	0.324
	Hours played (per day)	0.070	0.070	0.999	0.070	0.070 (0.935, 1.230)			
	Intercept	– 0.429	2.731	0.025	0.875	0.651			
Wrist									
1	Age (years)	0.054	0.032	2.882	0.090	1.055 (0.992, 1.123)	0.047	0.067	0.151
	Practice time (years)	0.104	0.037	8.018	0.005*	1.110 (1.033, 1.192)			
	Weekly practice frequency	0.168	0.078	4.596	0.032*	1.183 (1.015, 1.379)			
	Intercept	– 3.688	0.865	18.173	< 0.001*	0.025			

S.E.: standard error; %: percentage; C.I.: confidence interval. *Statistical significance for multiple regression analysis (*p* < 0.05).

### ROC curve

**Fig. 1:**
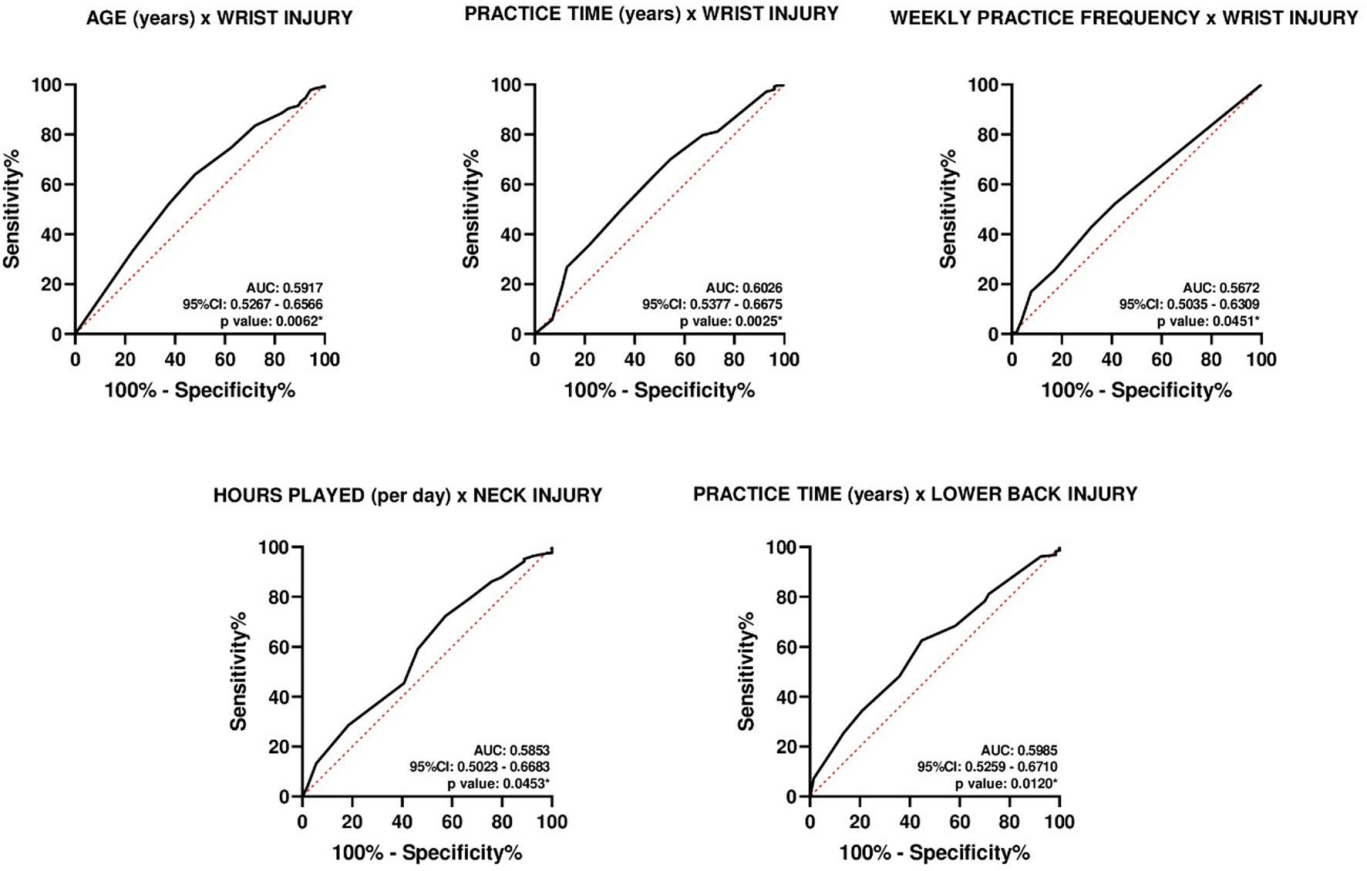
Receiver operating characteristic curve analysis to evaluate the discriminative power of age, practice time, weekly frequency, and daily hours in identifying musculoskeletal injuries. AUC: area under curve; %: percentage; CI: confidence interval. *The AUC was significantly different from 0.05.

According to the ROC curve analysis (Fig. 1), significant associations were observed for the following variables: age (years) with wrist injury (95% CI: 0.5267–0.6566; *p* < 0.0062), practice time (years) with wrist injury (95% CI: 0.5377–0.6675; *p* < 0.0025), weekly practice frequency with wrist injury (95% CI: 0.5035–0.6309; *p* < 0.0451), hours played (per day) with neck injury (95% CI: 0.5023–0.6683; *p* < 0.0453), and practice time (years) with low back injury (95% CI: 0.5259–0.6710; *p* < 0.0120). However, optimal cutoff values to achieve ideal sensitivity and specificity were not identified, due to the low discriminative capacity of the ROC curve (AUC < 0.70).

## Discussion

The aim of this study was to analyze the prevalence of injuries among esports players in Brazil, considering their distribution by body region, and to investigate factors associated with injury according to the affected area. The main findings were: (1) the wrist, low back, hands, and fingers accounted for the highest proportion of injury prevalence among Brazilian esports players; (2) esports practice time (in years) was associated with wrist injury; (3) weekly practice frequency was also associated with wrist injury; and (4) the model demonstrated a low discriminatory power for injury occurrence by body region.

### Injury prevalence

The prevalence of musculoskeletal pain observed in our study was similar to that reported by Difrancisco-Donoghue et al.^4^, who found a prevalence of 41% in the cervical and lumbar regions among collegiate esports athletes. These findings are also partially consistent with those of the systematic review by Tholl et al.^27^, which investigated musculoskeletal disorders in video game players. Regarding the wrist, hand, and finger regions, our findings are in line with the results of Kurniawan et al.^28^ e Clements et al.^29^, who studied professional and casual mobile esports players and collegiate electronic sports athletes, respectively, and reported a higher frequency of pain symptoms in these segments.

In addition to the distal regions of the upper limbs, other body areas, such as the shoulders and elbows may also be affected by esports practice, as evidenced by our results and supported by previous findings^28,29^. Musculoskeletal pain, therefore, emerges as a frequent symptom among esports practitioners. For instance, Lindberg et al.^30^, found that 42% of a sample of 188 Danish esports athletes reported musculoskeletal pain related to their practice.

Regarding training or practice load, our data showed a daily average of 5.83 ± 3.38 h played by men and 4.63 ± 3.03 h by female, with a median of 5 h and an interquartile range of 3 to 7 h. These findings are consistent with those reported by Clements et al.^29^ and Kurniawan et al.^28^, who also documented prolonged daily exposure to esports among their participants. This high number of hours may be partially explained by the demands of competitive performance. Players involved in school or university teams often engage in group training sessions lasting 3 to 4 h per day, followed by additional individual practice at home. However, this extended practice time frequently occurs in suboptimal environments, characterized by poor lighting, potentially inadequate postures, and low awareness of time, which may contribute to the onset or worsening of musculoskeletal dysfunctions^4^.

In addition to daily exposure time, the weekly practice frequency was also high in our sample, with a median of 6 days and an interquartile range of 4 to 8 days per week. This pattern is similar to that observed by Clements et al.^29^ in collegiate varsity electronic sports athletes, further highlighting the high level of involvement with esports among the participants assessed.

Another relevant aspect is the duration of esports experience, which also deserves attention. Although this variable has been scarcely explored in the literature, it was reported by Lam et al.^31^, who observed a median of 6 months of practice, with an interquartile range of 3 to 11 months among esports players. In our study, practice time proved to be a relevant factor, as it was associated with an increased risk of wrist injuries, suggesting that prolonged exposure to esports may contribute to the development of musculoskeletal dysfunctions, particularly in more heavily used body segments such as the upper limbs.

The exposure time, training intensity, and volume characteristics observed in the present study, along with training profiles reported in other studies, support the classification of upper limb injuries reported by participants as “overuse” injuries^32^. This type of injury is characterized by excessive training exposure without adequate recovery periods, which may hinder the necessary musculoskeletal adaptations required to meet the specific demands of the sport^32,33^.

It is important to note that although most esports are performed in a seated position, typically associated with sedentary behavior, the movements required from the wrist and hand are highly repetitive and frequent^34^. Notably, these movements are executed by smaller muscle groups with reduced cross-sectional area compared to those used in conventional sports^35^. As a result, these muscle groups may be more susceptible to fatigue, potentially leading to compensatory wrist and hand movements that favor the development of injuries and pain-related complaints^35,36^.

When analyzing the prevalence of musculoskeletal injuries stratified by sex, it is still not possible to accurately determine which group is more vulnerable to developing esports-related injuries. To date, the available literature has not conducted comparative analyses of injury occurrence between male and female players. However, previous studies have reported a higher frequency and prevalence of musculoskeletal injuries and/or pain among male players^28–30^, a finding that aligns with the results observed in our sample. However, the sample size between men and female was discrepant. In our analysis, we aimed to investigate whether sex could act as a factor associated with injury occurrence by body region; however, no statistically significant associations were found.

### Risk factors

The upper limbs are heavily engaged in esports practice due to the continuous execution of fine and repetitive motor movements over extended periods, as well as the prolonged maintenance of static postures^36^. One indicator of this demand is that while a novice esports player performs an average of around 50 actions per minute, elite-level esports athletes may execute between 500 and 600 movements per minute^34^.

The high demands placed on the upper limbs in esports may be influenced by several factors, including the type of game played. Among the potential risk factors for injury development, the influence of specific physical demands imposed by different game genres stands out^12^. For example, First-Person Shooter (FPS) players perform faster movements and cover greater distances across a wide, oblong mousepad area. In contrast, Multiplayer Online Battle Arena (MOBA) players exhibit a high number of hand direction changes, but these are concentrated within a smaller area. Adventure game players, on the other hand, display lower acceleration magnitudes, shorter distances covered, and fewer directional changes, although they also operate within a wide area similar to that of FPS players^12^. These genre-specific differences highlight the importance of understanding the underlying biomechanical and kinematic patterns to better identify musculoskeletal injury risk factors in esports players. However, the lack of stratification by different game genres in our study may have limited the ability to detect stronger associations and may partly explain the low explanatory power of the model, which accounted for approximately 4.7% to 6.7% of the variance in the dependent variable. Different esports genres generate different biomechanical and postural demands^12^, which can lead to heterogeneous injury patterns among players.

In addition to biomechanical demands and exposure time, inadequate ergonomics emerges as another factor potentially associated with the onset of injuries in esports players^30^. Specifically, regarding the wrist region, tasks such as typing can cause alterations to the median nerve, influenced by the degree of ulnar deviation. However, there is still no conclusive evidence that such alterations result in persistent symptoms or long-term nerve injuries^37^. Although the systematic review by Frutiger et al.^38^ indicated that customized workstation adjustments may reduce neck pain in office workers, the evidence presented was of low methodological quality, which limits its applicability to specific contexts such as esports. In this regard, beyond ergonomic modifications, other approaches may be more effective in reducing musculoskeletal symptoms. For example, in office workers, physical training as a treatment strategy has shown greater long-term promise compared to isolated ergonomic interventions^39^.

Moreover, it is important to recognize that extrinsic factors, such as inadequate ergonomics, do not act in isolation. Aspects such as physical activity level and sedentary time should also be considered potential risk factors. Even when partially offset by regular physical activity, sedentary behavior is associated with long-term adverse health outcomes^40^ and a higher incidence of musculoskeletal pain^41^. In a sample of 65 collegiate varsity esports players, 40% reported engaging in no physical activity outside of electronic gaming, and 15% remained seated for periods ≥ 3 h without breaks^4^. Similarly, Kurniawan et al.^28^ found that 76.6% of 94 participants (43 professional gamers and 51 casual gamers) exhibited low physical activity levels, as measured by the International Physical Activity Questionnaire. Conversely, the study by Lindberg et al.^30^, involving 188 Danish esports athletes, reported that participants met the physical activity recommendations proposed by the American Heart Association and the American College of Sports Medicine, suggesting 150 min of moderate intensity aerobic activity or 75 min of vigorous intensity aerobic activity per week, which can be distributed throughout the week and moderate intensity aerobic physical activity for a minimum of 30 min on five days per week, or vigorous intensity aerobic activity for a minimum of 20 min on three days per week, respectively^42,39^. Furthermore, it is noteworthy that the occurrence of repetitive movements, sustained over long periods during training sessions and competitions, is an intrinsic characteristic of esports modalities^31^. Consequently, esports practitioners and professional athletes require training of the physical capacities necessary to meet their specific demands, in order to mitigate the detrimental effects resulting from continuous exposure to repetition^8^. In addition to the harmful effects of continuous exposure to repetition, reduced rest periods impair the musculoskeletal system’s ability to recover, which, in turn, may hinder physical recovery and exacerbate the subjective perception of pain and dysfunction^.^

It is important to emphasize that the risk factors identified in the present study do not contribute to the onset of new injuries or the aggravation of pre-existing ones in isolation, but rather through the combined and complex interaction of multiple risk factors^41^. Injuries have a multifactorial nature and, consequently, require multidisciplinary interventions to address the demands presented by esports practitioners^4,8^. In this regard, combined interventions such as ergonomic adjustments, muscle strengthening and fatigue-resistance protocols, strategies aimed at optimizing sleep, and structured routine organization, may serve as valuable resources for both the prevention and rehabilitation of injuries^8^.

The ROC curve analysis revealed significant associations between specific player characteristics, such as age, cumulative practice time, weekly practice frequency, and daily hours played, and the occurrence of musculoskeletal injuries in our results, including wrist, neck, and lower back injuries. Despite these associations, the discriminative capacity of the analyzed variables was limited, as indicated by AUC values below 0.70, preventing the identification of reliable cutoff points for risk stratification. To our knowledge, no previous study has explored the discriminative capacity of these associations in esports players. These findings suggest that other factors, not included in our comprehensive and holistic assessment, likely contribute to injury risk, emphasizing the multifactorial nature of musculoskeletal injuries in competitive gaming esports. Future studies should investigate additional potential predictors, including biomechanical, ergonomic, and psychosocial variables, to evaluate their discriminative capacity and improve risk stratification strategies.

### Clinical impact

The findings of this study have relevant clinical implications for the management and prevention of musculoskeletal injuries in esports players. Practice time (in years), as well as the weekly frequency of engagement and/or training in electronic games, emerged as potential factors associated with an increased risk of wrist injuries. These results highlight the need for individualized strategic measures aimed at preventing musculoskeletal disorders and promoting health. One possible approach includes encouraging regular physical activity and/or implementing physical training programs tailored to the specific demands of esports, with the goal of enhancing performance and optimizing physical recovery when necessary^8,43^ especially considering that injuries associated with esports practice remain poorly understood and insufficiently investigated^42^. Additionally, esports-specific physical conditioning programs [46], ergonomic education strategies, structured rest, and proper sleep hygiene may play a significant role in the prevention and management of musculoskeletal pain among esports players^8^.

## Limitations

Although this study included a relevant sample size, the results should be interpreted with caution due to some limitations: (1) the findings are restricted to the population of Brazilian esports players; (2) the cross-sectional design precludes causal inferences; (3) data were obtained through a self-reported form, which may introduce recall bias; (4) the sample was not stratified by level of esports involvement (e.g., recreational vs. competitive; amateur vs. professional athletes); (5) the type or category of electronic game played was not considered; (6) The definition of injury adopted in this study considered only cases that resulted in absence from esports activities (time-loss). Limiting the definition of injury to cases that require medical attention or time off work may minimize the true impact of musculoskeletal problems, since less serious or overuse conditions often do not result in time off work, but may lead to a reduction in the volume or intensity of training^44^.

## Conclusion

The upper limbs were the most frequently affected segments by injuries among Brazilian esports players, with the highest prevalence observed in the wrists, hands, and fingers, followed by the low back, which was the second most affected body region. Logistic regression analysis identified years of practice and weekly frequency as risk factors for wrist injury, explaining between 4.7% and 6.7% of the variance in the dependent variable.

## Data Availability

The datasets used and/or analyzed during the current study are available from the corresponding author on reasonable request.
